# Community stakeholder preferences for evidence-based practice implementation strategies in behavioral health: a best-worst scaling choice experiment

**DOI:** 10.1186/s12888-021-03072-x

**Published:** 2021-02-04

**Authors:** Nathaniel J. Williams, Molly Candon, Rebecca E. Stewart, Y. Vivian Byeon, Meenakshi Bewtra, Alison M. Buttenheim, Kelly Zentgraf, Carrie Comeau, Sonsunmolu Shoyinka, Rinad S. Beidas

**Affiliations:** 1 School of Social Work, Boise State University, Boise, ID USA; 2 Department of Psychiatry, University of Pennsylvania Perelman School of Medicine, Philadelphia, PA USA; 3 Leonard Davis Institute of Health Economics, University of Pennsylvania, Philadelphia, PA USA; 4 Department of Psychology, University of California, Los Angeles, Los Angeles, CA USA; 5 Center for Clinical Epidemiology and Biostatistics, Perelman School of Medicine, University of Pennsylvania, Philadelphia, PA USA; 6 Division of Gastroenterology, University of Pennsylvania, Philadelphia, PA USA; 7 Department of Biostatistics and Epidemiology, University of Pennsylvania, Philadelphia, PA USA; 8 Department of Medical Ethics and Health Policy, Perelman School of Medicine, University of Pennsylvania, Philadelphia, PA USA; 9 Center for Health Incentives and Behavioral Economics, University of Pennsylvania, Philadelphia, PA USA; 10 Department of Family and Community Health, School of Nursing, University of Pennsylvania, Philadelphia, PA USA; 11 Department of Behavioral Health and Intellectual disAbility Services (DBHIDS), Philadelphia, PA USA; 12 Department of Medicine, Perelman School of Medicine, University of Pennsylvania, Philadelphia, PA USA; 13 Penn Implementation Science Center at the Leonard Davis Institute of Health Economics (PISCE@LDI), University of Pennsylvania, 3535 Market Street, 3015, Philadelphia, PA 19104 USA

**Keywords:** Evidence-based practice, Implementation, Stakeholder preferences, Participatory design

## Abstract

**Background:**

Community behavioral health clinicians, supervisors, and administrators play an essential role in implementing new psychosocial evidence-based practices (EBP) for patients receiving psychiatric care; however, little is known about these stakeholders’ values and preferences for implementation strategies that support EBP use, nor how best to elicit, quantify, or segment their preferences. This study sought to quantify these stakeholders’ preferences for implementation strategies and to identify segments of stakeholders with distinct preferences using a rigorous choice experiment method called best-worst scaling.

**Methods:**

A total of 240 clinicians, 74 clinical supervisors, and 29 administrators employed within clinics delivering publicly-funded behavioral health services in a large metropolitan behavioral health system participated in a best-worst scaling choice experiment. Participants evaluated 14 implementation strategies developed through extensive elicitation and pilot work within the target system. Preference weights were generated for each strategy using hierarchical Bayesian estimation. Latent class analysis identified segments of stakeholders with unique preference profiles.

**Results:**

On average, stakeholders preferred two strategies significantly more than all others—compensation for use of EBP per session and compensation for preparation time to use the EBP (*P* < .05); two strategies were preferred significantly less than all others—performance feedback via email and performance feedback via leaderboard (*P* < .05). However, latent class analysis identified four distinct segments of stakeholders with unique preferences: Segment 1 (*n* = 121, 35%) strongly preferred financial incentives over all other approaches and included more administrators; Segment 2 (*n* = 80, 23%) preferred technology-based strategies and was younger, on average; Segment 3 (*n* = 52, 15%) preferred an improved waiting room to enhance client readiness, strongly disliked any type of clinical consultation, and had the lowest participation in local EBP training initiatives; Segment 4 (*n* = 90, 26%) strongly preferred clinical consultation strategies and included more clinicians in substance use clinics.

**Conclusions:**

The presence of four heterogeneous subpopulations within this large group of clinicians, supervisors, and administrators suggests optimal implementation may be achieved through targeted strategies derived via elicitation of stakeholder preferences. Best-worst scaling is a feasible and rigorous method for eliciting stakeholders’ implementation preferences and identifying subpopulations with unique preferences in behavioral health settings.

**Supplementary Information:**

The online version contains supplementary material available at 10.1186/s12888-021-03072-x.

## Background

The need to improve the quality and outcomes of health and behavioral health services has led to increased emphasis on the implementation of evidence-based practices (EBPs) in community settings [1–4]. Effective implementation of EBPs requires the cooperation of clinicians, supervisors, and administrators who deliver clinical care. However, little is known about these stakeholders’ values and preferences for specific types of implementation strategies, defined as the active approaches used to improve adoption, implementation, and sustainment of EBPs [5]. It is also not clear how best to elicit, quantify, and segment stakeholders’ implementation preferences. Community stakeholder preferences should be considered when selecting implementation strategies for several reasons. First, the process of eliciting preferences is, in and of itself, a way to increase stakeholder engagement and buy-in, a key component of the implementation process [6–8]. Second, there is evidence that tailored implementation strategies (i.e., those that address localized barriers) are more effective than non-tailored strategies [9, 10] and stakeholder preferences may provide insights regarding how to tailor to local contexts [9]. Third, because stakeholder preferences may not align with evidence on what works, understanding preferences is an essential first step in determining where implementation efforts should start in terms of targeted mechanisms of change.

To date, efforts to elicit stakeholder implementation preferences using both qualitative and quantitative approaches have had several limitations. Qualitative interviews are useful for generating deep understanding among a small group; however, they are resource intensive and may have limited generalizability. Recent advances in quantitative measurement include pragmatic Likert-type scales that allow stakeholders to rate the acceptability, feasibility, and appropriateness of implementation strategies [11]. These approaches are relatively low-cost even for large samples; however, because they do not require respondents to consider trade-offs, they typically suffer from strong ceiling effects with many strategies ending up highly-ranked, thus undermining their utility.

Stated preference choice experiments are a promising set of methods for eliciting stakeholder preferences that may overcome these limitations by engaging stakeholders in an intuitive yet powerful set of choice tasks that closely mimic real-life decisions and that can be easily implemented in large samples [12]. By requiring respondents to consider trade-offs across a set of choices, choice experiments generate highly-accurate estimates of implicit preferences for a targeted set of objects (e.g., implementation strategies) in a time-efficient, cost-effective, and generalizable manner [12–15]. These methods are especially valuable when the set of objects are carefully derived through elicitation work within the target population and when information on actual behavior or decisions are unavailable (or unobtainable), as is typically the case in implementation [16].

Best-worst scaling (BWS) [16, 17] is a type of choice experiment uniquely suited to the task of eliciting implementation preferences. This is because BWS is flexible enough to identify either (a) the most preferred strategy(s) from a list of irreducible and dissimilar strategies, or (b) the most preferred level (e.g., dollar amount) of an attribute (e.g., compensation) that multiple strategies have in common [17]. This is important because there are 73 discrete implementation strategies which can be combined in many permutations [18]. Second, respondents’ BWS choices can be segmented using model-based clustering procedures such as latent class analysis to identify subpopulations that share similar preferences [19, 20]. Segmentation allows planners to optimally target implementation strategies to subpopulations based on their preferences and therefore potentially optimize their impact.

The goals of this study were to apply BWS to (1) characterize and quantify the preferences of clinicians, supervisors, and administrators employed within clinics that deliver publicly-funded behavioral health services for a set of 14 implementation strategies, (2) empirically identify segments of stakeholders that exhibit distinct preferences, and (3) examine differences across segments in professional characteristics (e.g., age, education, primary role in organization).

## Methods

### Setting

Philadelphia, a city of over 1.5 million residents, is the poorest of the United States’ 10 largest cities (26% of residents live below the poverty level) [21, 22]. The city’s population is 41% African-American, 35% Non-Hispanic White, 15% Hispanic, 8% Asian, and 2% other race [22, 23]. Public behavioral health services (i.e., mental health and substance use treatment) in Philadelphia are financially supported by Medicaid and managed by Community Behavioral Health (CBH), a non-profit managed care organization (i.e., “carve-out”) established by the city that functions as a component of the Department of Behavioral Health and Intellectual disAbility Services (DBHIDS). In 2018, DBHIDS and CBH included 175 in-network provider organizations serving 118,011 unique members [24].

Since 2007, DBHIDS has supported EBP delivery in Philadelphia through a series of “EBP initiatives” that include training, expert consultation, and implementation supports (e.g., booster trainings, implementation meetings) for participating clinicians [25]. These initiatives have supported implementation of several cognitive behavioral therapy models including cognitive therapy, prolonged exposure, trauma-focused cognitive-behavioral therapy, dialectical behavior therapy, and parent child interaction therapy for a range of psychiatric disorders. In 2013, DBHIDS created a centralized infrastructure called the Evidence-based Practice and Innovation Center (EPIC) to oversee EBP implementation efforts. EPIC supports EBP implementation by coordinating and consulting EBP efforts across the clinics within the CBH network (the managed care organization), contracting with treatment experts to deliver EBP training, contracting with treatment providers to deliver EBP, providing EBP consultation and implementation support, hosting events to publicize EBP delivery, maintaining web-based resources (e.g., webinars), designating EBP programs within provider agencies, and providing financial incentives (e.g., enhanced rates) for delivery of EBPs.

### Participants

The target population for this study was clinicians, supervisors, and administrators employed within clinics that deliver publicly-funded behavioral health services in the City of Philadelphia. The sample did not include members of EPIC (i.e., it did not include treatment experts or consultants). Because DBHIDS does not maintain a roster of email addresses to directly contact active clinicians, we used a two-pronged recruitment and sampling approach. We sent invitation emails to leaders of behavioral health organizations (*n* = 210), clinicians (*n* = 527), and other community stakeholders (e.g., directors of a clinician training organization; *n* = 6) in Philadelphia. We also e-mailed the invitation to four local electronic mailing lists known to reach large swaths of the CBH network (e.g., managed care organization listserv) and asked organization and network leaders to forward the email. From these contacts, the survey link was opened 654 times and 343 respondents completed the BWS choice experiment.

### Study design and procedure

The BWS choice experiment was designed to quantify stakeholders’ preferences for 14 implementation strategies developed through iterative elicitation, pilot, and pre-testing work completed with members of each stakeholder group in the target population [17, 26]. Elicitation of strategies was completed via a system-wide innovation tournament, described elsewhere [27], through which clinicians submitted ideas for strategies to support EBP implementation in Philadelphia. Following the tournament, submitted ideas (*N* = 65) were analyzed and refined by a team of implementation scientists, behavioral scientists, and clinicians, in order to develop a set of distinct, clearly operationalized implementation strategies with ecological validity for the target system. The analysis process involved combining similar strategies, crafting definitions of each strategy, and ensuring that all strategies were adequately captured by the final set. This process resulted in a set of 14 implementation strategies (see Table 1: *List of Implementation Strategies Included in the BWS Experiment*), which were subsequently evaluated in pre-testing interviews with clinicians, supervisors, and administrators (*n* = 9) within the system to ensure that the strategies, as described, spanned the full range of approaches viewed as relevant by stakeholders and were clearly described. The 14 strategies fell into six categories: (1) financial incentives, (2) clinical consultation, (3) clinical support tools, (4) clinician social support and networking, (5) clinician performance feedback/social comparison, and (6) client supports [27]. Notably, the strategies developed through this process addressed 8 out of 9 categories of implementation strategies identified in the Expert Recommendations for Implementing Change (ERIC) project [18], including: use evaluative and iterative strategies, provide interactive assistance, develop stakeholder interrelationships, train and educate stakeholders, support clinicians, engage consumers, utilize financial incentives, and change infrastructure. Supplemental Table 1A in Additional File 2 shows how the strategies from the present study aligned with the discrete implementation strategies identified by the ERIC project.

**Table 1:** List of Implementation Strategies Included in the BWS Choice Experiment

Category	Strategy Name	Definition
Financial Incentives	EBP certification bonus	Receipt of a 1-time bonus for verified completion of a certification process over a 1-year period, in which clinicians: attend four, 1-day booster training sessions; pass a multiple-choice knowledge test; and submit one tape of a session with a client where they use the EBP.
	Compensation for use of EBP per session	Receipt of additional compensation (in addition to regular paycheck) upon verification of using the EBP in sessions with clients for whom it is appropriate (i.e., per session), up to a specified amount per year.
	Compensated time for EBP preparation	Ability to bill for any verified time clinicians spend preparing to use the EBP (e.g., reviewing the protocol, preparing materials for session, reviewing client homework, etc.), up to a specified amount per year.
Clinical Consultation	Expert-led EBP consultation	1-h, monthly, web- or phone-based consultation, with up to 5 other clinicians, for 1 year led by an expert EBP trainer.
	Peer-led EBP consultation	1-h, monthly web- or phone-based conference, with up to 5 other clinicians, for 1 year led by a clinician with experience implementing the EBP in Philadelphia.
	Expert in your back pocket (on call)	Network of EBP trainers on call via phone or web chat for same-day, 15-min consultations to problem-solve issues with implementing the EBP.
Clinical Support Tools	Web-based resource center/ mobile app	Includes: (a) video examples of how to use specific techniques for the EBP, (b) “session checklists” with steps outlined for using the EBP techniques in session, and (c) downloadable worksheets and measures needed to use the EBP.
	Electronic evidence-based screening instrument inventory	Evidence-based screening instruments included in an electronic medical record, completed electronically by clients in the waiting room (e.g., tablet); results are automatically scored and immediately available so clinicians can assess treatment needs and track client progress.
Clinician Social Support and Networking	EBP-focused online forum	Confidential site available only to registered clinicians who use the EBP, where clinicians can login and post questions and answers about using the EBP, share tips, and identify resources for using the EBP.
	Community-based EBP mentoring program	One-on-one mentoring program, where clinicians are matched with a local peer clinician who works with the same population to support each other in implementing the EBP.
Performance Feedback / Social Comparison	EBP Performance benchmark leaderboard	Posted where only agency staff can view it, recognizing clinicians in the agency who met a benchmark for EBP implementation each month (based on 3 randomly selected sessions).
	EBP Performance benchmark email	Available only to the clinician and his/her supervisor, reporting whether s/he met a benchmark for EBP implementation each month (based on 3 randomly selected sessions).
Client Supports	Client mobile app/ texting service	Provides clients with reminders to attend sessions, prompts to complete homework assignments, and clinician-tailored messages about practicing EBP skills.
	Improved waiting room	Create a relaxing waiting room (e.g., physical appearance, sensory experience) that helps prepare the client to enter the session ready to work on EBP content.

Because each of the 14 strategies represented a qualitatively unique strategy, we used object case BWS (as opposed to profile case or multi-profile case BWS) [28]. The BWS experimental design was generated using the Sawtooth Discover algorithm which produces randomized choice sets with optimal frequency balance, orthogonality, positional balance, and connectivity for a given sample size [29–33]. Within the design, each participant was shown 11 sets of four randomly selected and randomly ordered strategies and, within each set, asked to choose which strategy was “Most useful” (i.e., best) for supporting clinicians’ implementation of psychosocial EBPs and which strategy was “Least useful” (i.e., worst). The Discover algorithm optimizes 1-way, 2-way, and positional balance within the randomization sequence such that (a) each strategy is presented an equal number of times, (b) each pair of strategies appears in a set an equal number of times, and (c) each strategy is shown in each position an equal number of times. For this study, each strategy was included in at least three sets. Participants were instructed to imagine that their organization had decided to adopt a new psychosocial EBP that exhibited excellent outcomes for their specific client population, and that this treatment was new to the respondent (or to clinicians working in the respondent’s setting; see Additional File 1 for the BWS prompt and an example set of strategies). The prompt explained that initial training in the EBP would be provided and would include active learning approaches, and their input was sought regarding the best implementation strategies that could be used to support clinicians’ implementation of the new practice following training.

Sample size calculations assumed an alpha level of .05, margin of error of 0.1, and 14 implementation strategies to be rated with each strategy appearing in a minimum of 3 sets. Based on these assumptions, the required sample size was *N* = 244 participants rating 11 sets of 4 strategies each [28, 34].

The BWS experiment was implemented via a web-based computerized survey emailed to clinicians, supervisors, and administrators from March 2019 to April 2019. Consistent with best practices in survey administration, we utilized a process [35] in which participants received a pre-survey priming email, survey invitation email, and three follow-up reminders, delivered approximately 1 week apart. Surveys took approximately 30 min and participants received a $25 gift card.

### Measures

In addition to completing the BWS questions, respondents reported on professional and workplace characteristics: primary role (administrator [those who were executive level administrators within the clinics], supervisor [those who supervise clinicians in clinical work], clinician [those who primarily offer direct services to clients]), education level, type of clinic in which they were employed (mental health, substance use, dual diagnosis), salary versus fee-for-service employment, tenure in current agency, years of experience as a clinician, extent to which their graduate training emphasized EBP (ranging from 1 = *Never* to 7 = *Always*), average hours worked per week, number of City-sponsored EBP training initiatives in which the respondent had participated (ranging from 0 to 6), number of BWS strategies currently in use by their employing agency (ranging from 0 to 14), age, sex, race, and ethnicity. Because of heterogeneity across roles, administrators and supervisors did not report on salary versus fee-for-service employment, hours worked per week, extent to which their graduate training emphasized EBP, years of experience as a clinician, or number of City-sponsored EBP training initiatives participated in.

### Data analysis

Best and worst choice frequencies for each strategy were summarized at the sample level using count analysis which represents the proportion of times a strategy was chosen as most or least useful relative to the number of times it was displayed [17]. Preference weights for each strategy were calculated at the individual level using hierarchical Bayes estimation with a multinomial logit model implemented using CBC/HB software from Sawtooth (version 5) [36–40]. Latent class analysis (LCA) [19, 20, 41] was used to identify segments of the population with different preferences and to estimate preference weights (i.e., part worth utilities) for each segment using Sawtooth Software’s LCA program (version 4.7), which implements the estimation procedure described by DeSarbo and colleagues [19]. We estimated LCA models with 1 through 5 classes. Consistent with best practices, we selected the best-fitting model on the basis of the Bayesian information criterion [42], probabilities of correct classification [43], sufficiently populated classes, and interpretability of classes based on alignment with previous research and theoretical considerations [44]. Differences across segments on professional characteristics were tested using analyses of variance and chi-square tests (SPSS, Version 25). There were no missing data on participants’ preferences. Because very few participants (< 5%) had missing data on professional and sociodemographic variables, these were excluded from analyses on a pairwise basis.

## Results

Participants were 76% female. With regard to ethnicity and race, participants endorsed the following categories: White (60%), Black and/or African American (20%), American Indian or Alaskan Native (1%), Asian (3%), Other (7%). The remainder were missing or preferred not to disclose. Participant demographics are largely consistent with previous work we have conducted in the city of Philadelphia [45] and broader national trends [46].

**Fig. 1:**
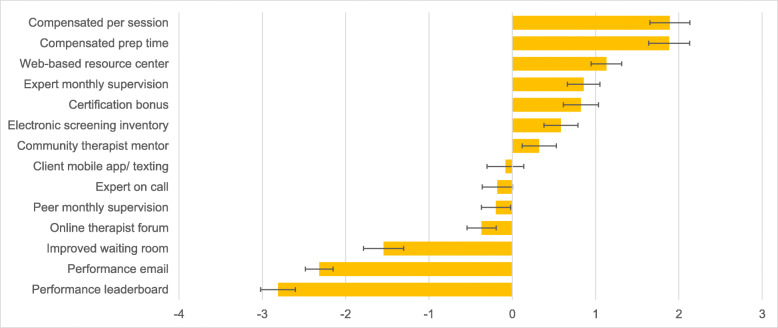
Average Preference Weights for each Strategy (*N* = 343) *Note:* Preference weights (i.e., part worth utilities) were estimated via hierarchical Bayes estimation incorporating a multinomial logit model. Values are logit scaled; strategies with higher preference weights are more preferred. Error bars indicate 95% confidence intervals. When 95% confidence intervals do not overlap between two strategies, the strategy with the higher value is significantly more preferred at *p* < .05

Table 2 shows the best and worst choice frequencies for each strategy. Fig. 1 shows the mean preference weights (i.e., part worth utilities) for each strategy with 95% confidence intervals. The preference weights are logit scaled and represent the average utility or value that this sample of respondents attached to each strategy; higher values indicate greater utility. When 95% confidence intervals do not overlap between two strategies, the strategy with the higher value is significantly more preferred at *p* < .05. The two strategies viewed as most useful were both within the financial incentives category and included (1) compensation for EBP use per session and (2) compensation for EBP preparation time. Both of these were preferred significantly more than all other strategies (see Fig. 1). Conversely, both performance feedback/social comparison strategies were viewed as significantly less useful than all others (see Fig. 1): (1) performance feedback via leaderboard was the least preferred, followed by (2) performance feedback via email. On average, financial incentive strategies were preferred 9.2 times more than performance feedback/social comparison strategies (Mean Best = .46 vs. .05) and performance feedback/social comparison strategies were disliked 5.1 times more than financial incentive strategies (Mean Worst = .56 vs. .11).

**Table 2:** Sample Best and Worst Choice Frequencies

Implementation Strategy	B	W	B - W	# of times displayed
Compensated per session	0.46	0.10	0.36	1079
Compensated prep time	0.45	0.11	0.35	1079
Web-based resource center	0.36	0.12	0.24	1084
Expert monthly supervision	0.32	0.15	0.18	1094
Certification bonus	0.34	0.17	0.17	1074
Electronic screening inventory	0.31	0.18	0.13	1075
Community clinician mentor	0.27	0.20	0.08	1079
Client mobile app/ texting	0.22	0.24	−0.02	1076
Peer monthly supervision	0.18	0.23	−0.05	1080
Expert on call	0.19	0.24	−0.05	1080
Online therapist forum	0.18	0.26	−0.08	1081
Improved waiting room	0.12	0.41	−0.29	1070
Performance email	0.05	0.52	−0.47	1076
Performance leaderboard	0.04	0.59	−0.55	1065

*N* = 343. B = sample-level best choice frequency calculated as the proportion of times the strategy was selected as “Most Useful” relative to the number of times it was displayed; W = sample-level worst choice frequency calculated as the proportion of times it was selected as “Least Useful” relative to the number of times displayed. B – W = best minus worst scores calculated as proportion best less proportion worst

**Fig. 2:**
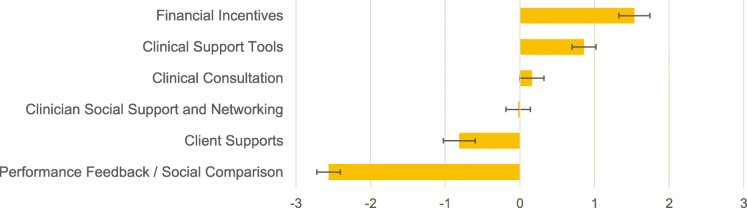
Average Preference Weights for each Category. *Note:* Preference weights (i.e., part worth utilities) were estimated via hierarchical Bayes estimation incorporating a multinomial logit model. Categories with higher average preference weights are more preferred. Error bars indicate 95% confidence intervals. When 95% confidence intervals do not overlap between two categories, the category with the higher value is significantly more preferred at *p* < .05. See Table 1 for the specific strategies included in each category

Additional insight into stakeholders’ preferences can be obtained by examining their preferences grouped by the six categories of strategies. As is shown in Fig. 2, strategies in the financial incentives category were preferred significantly more on average than all others (*p* < .05), followed by clinical support tools, which were the second most preferred and rated significantly higher than all others except financial incentives (*p* < .05). The clinical consultation and social networking categories were statistically indistinguishable but rated significantly higher than client supports which, in turn, rated significantly higher than performance feedback/social comparison.

**Fig. 3:**
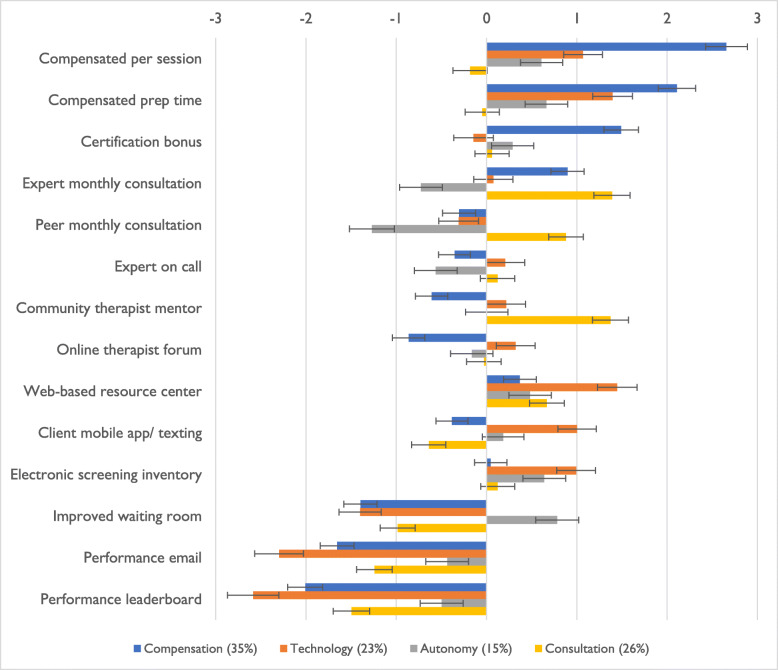
Preference Weights by Latent Class Segment. *Note: N* = 343. Segments and preference weights (i.e., part worth utilities) derived via latent class analysis. Values are logit scaled; strategies with higher preference weights are more preferred. Error bars indicate 95% confidence intervals. When 95% confidence intervals do not overlap between two strategies, the strategy with the higher value is significantly more preferred at *p* < .05. Segment labels reflect the type of implementation support prioritized by the segment relative to others. Segment 1, *Support me through Financial Incentives* (Compensation), included *n* = 121 participants; segment 2, *Support me through Technology*, included *n* = 80 participants; segment 3, *Support me through Autonomy*, included *n* = 52 participants; and segment 4, *Support me through Consultation*, included *n* = 90 participants

Figure 3 shows the preference weights (i.e., part worth utilities) and 95% confidence intervals for each strategy for each of the four segments identified in the optimally-fitting four-class LCA model. These preference weights are interpreted in the same manner as those shown in Fig. 1. Tables 3 and 4 (see Additional File 2) show the distribution of professional and sociodemographic characteristics by segment and for the full sample. Segment 1, labeled *Support Therapists through Financial Incentives,* included 35% of the sample (*n* = 121) and exhibited significantly higher preferences for compensation per session, compensation for preparation time, and compensation for certification compared to all other segments. Segment 1 had the highest proportion of administrators (17%, *n* = 20) relative to the other groups (3 to 5%, *p* = .006) (see Table 3 included as an additional file (see Additional file 2)).

Segment 2, labeled *Support Therapists through Technology,* included 23% of the sample (*n* = 80) and exhibited significantly higher preferences for the client mobile app/texting service and the web-based clinician resource center/mobile app compared to the other segments. This segment exhibited significantly less favorable preferences for the performance feedback email and performance leaderboard relative to other groups. Segment 2 tended to have fewer years of experience in their current agency (*p* = .061) and to be younger on average (*p* = .065).

Segment 3, labeled *Support Therapists through Autonomy,* included 15% of the sample (*n* = 52). This segment exhibited the only favorable rating of the improved waiting room strategy and these ratings were significantly higher than those of the other segments. This segment also exhibited significantly less preference for EBP consultation led by either experts or peers. Members of this segment exhibited lower than average participation in the EBP initiatives provided by the city (*p* = .021) and the fewest average hours worked per week (*p* = .009).

Segment 4, labeled *Support Therapists through Consultation,* included 26% of the sample (*n* = 90) and exhibited significantly higher preferences for expert-led monthly consultation, peer-led monthly consultation, and a community-based EBP mentoring program. This segment also exhibited significantly lower preferences than the other groups for compensation per session and compensation for preparation time. This segment had the highest proportion of clinicians (38%) who worked in clinics focused on the treatment of substance use disorders (*p* = .020), although similar to other segments, most in this group worked in clinics focused on the treatment of mental health disorders (62%).

## Discussion

This study provides valuable insights on clinician, supervisor, and administrator preferences for implementation planning in large public behavioral health systems and highlights important directions for future research. Results also illustrate the utility of BWS as a methodology for rigorously and efficiently eliciting stakeholder preferences for implementation strategies in large-scale behavioral health and health systems.

By identifying four distinct subpopulations of clinicians, supervisors, and administrators whose preferences reflected distinct foci for implementation strategies, these findings highlight the heterogeneity of stakeholder preferences and point to the need for a new research agenda that unpacks the relationships between preference, implementation effectiveness, and tailoring of implementation strategies. Even as Segment 1 (35% of the sample) strongly preferred all financial incentive strategies above any other strategy, another group, Segment 4 (26% of the sample), showed much less interest in financial incentives, preferring instead consultation with EBP experts, and yet another group, Segment 2 (23% of the sample) exhibited strong preferences for technology-based strategies. These groups were all distinct from Segment 3 (15% of the sample) which preferred an improved waiting room (to help relax patients and prepare them to engage in an EBP-focused session) and viewed any type of clinical consultation as least helpful. These distinct segments suggest that a one-size-fits-all implementation strategy may not be successful, and certainly will not be preferred, by the majority of stakeholders. Different implementation strategies may need to be matched with these distinct subpopulations. There is growing consensus in implementation science that strategies should be selected and tailored based on contextual factors with regard to the EBP, setting, and individual characteristics [47, 48]. Our results highlight stakeholder preference as a potentially important dimension for tailoring implementation strategies and point to the need for research to better understand how preferences influence EBP implementation.

A few prior studies have used related choice experiment methods such as discrete choice experiments to understand practitioners’ preferences for features of EBP training and their beliefs regarding variables that might influence their adoption of EBP [8, 10, 49]. The present study extends this prior work by focusing on stakeholders’ preferences for post-training implementation strategies drawn from a diverse set of categories that represent the majority of ERIC strategies (e.g., financial incentives vs. client supports vs. clinician social networking vs. performance feedback/social comparison). It is well-established that post-training support is typically necessary in order to generate sustained and meaningful change in practice behaviors [50]; our results provide insight into what types of post-training implementation strategies are viewed as most useful by stakeholders in community mental health as well as the heterogeneity in those preferences. In addition, by using BWS to directly compare multiple dissimilar types of strategies (e.g., financial incentives vs. client supports vs. clinician social networking, etc.), our results offer the first glimpse into stakeholders’ prioritization of these different categories. In some ways, our study provides a view of the forest (i.e., which categories of strategies do stakeholders most prefer?) which primes the field for future work, using discrete choice experiments, to identify stakeholders’ preferences for the design of specific strategies (i.e., trees). For example, discrete choice experiments could be fielded to determine stakeholders’ preferences for the specific features of any of the strategies included in our BWS choice experiment (e.g., the most preferred features of a system that compensates per session).

Targeting implementation strategies based on stakeholders’ preferences may result in more successful EBP implementation in at least three ways. First, *if* implementation strategies are differentially effective for different individuals and contexts, stakeholder preferences may signal which strategy will be most effective for a given situation. In this case, stakeholder preferences represent a valid signal indicating which strategy will be most effective in their specific circumstances and strategy effectiveness would be optimized by matching strategies to specific individuals or organizations based on the insights of participants. This theory assumes that stakeholders’ preferences are valid indicators of which strategy will work best which has not yet been empirically verified. This is similar to the idea of precision medicine in which the most effective intervention (i.e., implementation strategy) depends on the characteristics of the specific individual in context.

Second, targeting strategies to stakeholders’ preferences may have a general *accelerator effect* that increases the effectiveness of any strategy compared to its baseline effectiveness due to increased engagement or buy-in. For example, if participants are more engaged or invested in a strategy because they chose it, they may be willing to exert more effort to ensure its success and this may increase the strategy’s effectiveness. In this case, the act of choosing a preferred strategy is in itself an intervention that might improve implementation success. Ideally, research could quantify the magnitude of this ‘preference effect’ to determine how much increase in effectiveness could be expected for any strategy simply by allowing stakeholders to choose it.

Third, assuming that some strategies are universally more effective than others, it may be beneficial to understand stakeholders’ preferences so that policymakers and other leaders can identify stakeholders who do not prefer effective strategies and use supplemental interventions (e.g., a readiness strategy) with these individuals prior to, or concurrent with, the launch of the effective strategy. In this scenario, individual preferences have no accelerator effect on strategies’ effectiveness, nor do they provide a valid guide to the choice of strategy; rather, assessment of preferences allows system leaders to identify subpopulations of stakeholders who may benefit from supplementary interventions (e.g., pre-implementation strategy) to support their engagement with a system-selected, effective strategy that is going to be rolled out.

In contrast to the hypotheses described above, it may be that preference has no effect on the outcome of implementation strategies whatsoever. The identification of four distinct preference subpopulations in this study points to the need for research to determine how preferences relate to implementation effectiveness so that resources devoted to implementing EBPs can be optimized.

Across the full sample, one consistent finding was the overall rejection of performance feedback/social comparison strategies, which were rated lower than all other strategies on average for the full sample and were the lowest rated strategies for 3 out of 4 subpopulations. These findings suggest stakeholders overwhelmingly viewed performance feedback/social comparison strategies as unhelpful for supporting EBP implementation. This is consistent with findings from primary care practices, in which primary care clinicians also disliked strategies using social comparison [51]. Future qualitative inquiry could provide valuable insights into why stakeholders view feedback/social comparison strategies as unhelpful. Potential mechanisms include discomfort with receiving information that is misaligned with one’s perception of one’s performance or feeling as though there will be negative consequences for poor performance.

The large preference gap between feedback/social comparison and other strategies, such as financial incentives, which emerged as the most preferred strategy on average in the full sample and the first or second choice strategy for 3 out of 4 subpopulations, raises an important question about the relative effectiveness of high cost financial incentives compared to lower cost performance feedback strategies, both of which have some evidence of effectiveness [52, 53]. Comparative effectiveness trials that include cost-effectiveness analyses would aid policymakers in selecting among strategies when there is a mismatch between stakeholders’ preferences versus what is known to be effective [54]. The generally favorable view of financial incentives in this sample is not surprising against the backdrop of a publicly-funded behavioral health workforce that is poorly compensated and financially stressed, often employed as independent contractors, and are working within organizations that are also struggling financially [55, 56].

Another issue highlighted by these findings is the question of which stakeholder group’s preferences have the strongest implications for successful implementation. Many systems, such as Philadelphia, focus implementation policies primarily at the program level by designating EBP programs and providing financial incentives to agencies (versus clinicians). This raises the question of to what extent policymakers should focus their attention on the preferences of administrators, who make agency-level decisions about EBP (e.g., whether to respond to agency incentives by implementing an EBP program), versus attending to the preferences of clinicians, who ultimately are responsible for implementing EBPs in direct care. Preliminary evidence regarding pay-for-performance in behavioral healthcare suggests organization-focused financial incentives have minimal impact on practice whereas individual clinician-focused incentives can change provider behavior [53].

Our results are subject to limitations and qualifications. Stated preferences were elicited from a controlled experiment on hypothetical implementation options. Real-world implementation behaviors are complicated by numerous factors not accounted for in our controlled experiment; thus, actual implementation behavior could be different from that predicted by our data. However, several features of the study design were implemented following best practice methods and consequently limit the potential for bias. For example, the scenario and implementation strategies were presented as realistically as possible and the number of questions each respondent answered was limited considering the cognitive burden of choice questions. The use of object case BWS allowed us to estimate respondents’ preferences for a wide range of qualitatively distinct implementation strategies; however, this design choice sacrificed the opportunity to obtain finer-grained information about which features of specific strategies are most preferred (e.g., the design and amount of compensation for EBP use per session). Other types of choice experiments, such as discrete choice experiments and profile case BWS, generate fine-grained estimates of respondents’ preferences for specific levels of strategy features. Studies incorporating those approaches represent a potentially valuable extension of this work. The specific implementation preferences described by this sample of clinicians and administrators in this large public behavioral health system were limited by those generated through the system-wide innovation tournament. In addition, this sample’s preferences may not generalize to clinicians in more rural areas or in cities or states that have not exhibited similar support for EBP. Further, the particular set of strategies is likely tied to the structure of the US behavioral healthcare system and likely would not generalize to other countries with different healthcare systems. The use of a motivated volunteer sample of stakeholders, while preserving internal validity, may also limit generalizability and affect the relative proportions in the latent class analysis. Finally, clinician preferences are but one factor in many that should guide the selection of implementation strategies to support EBP in a specific setting.

## Conclusions

Effective implementation of EBP in health and behavioral health systems must include the active participation of stakeholders who receive, deliver, and/or oversee the delivery of clinical care. Numerous groups, including service participants, family members, clinicians, supervisors, administrators, funders, and policymakers, have a stake in implementation decisions and understanding their values and preferences for implementation strategies may be one way to increase stakeholder engagement and implementation effectiveness. Results from this study demonstrate the presence of four distinct subpopulations of clinicians, supervisors, and administrators whose implementation preferences differ and who may not all respond positively to a one-size-fits all implementation strategy. As such, these findings highlight the need for research on how stakeholder preferences intersect with implementation effectiveness and the tailoring of implementation strategies. Furthermore, this study demonstrates that BWS choice experiments are a highly feasible and rigorous method for eliciting stakeholders’ preferences regarding how to support their implementation of EBP.

## Supplementary Information

**Additional file 1.** The BWS prompt and an example set of strategies.

**Additional file 2.** Participant Characteristics Overall and by Preference Segment, shows the distribution of professional and sociodemographic characteristics by segment and for the full sample.

## Data Availability

Data will be made available upon request. Requests for access to the data can be sent to the Penn ALACRITY Data Sharing Committee. This Committee is comprised of the following individuals: Rinad Beidas, PhD, David Mandell, ScD, Kevin Volpp, MD, PhD, Alison Buttenheim, PhD, MBA, Steven Marcus, PhD, and Nathaniel Williams, PhD. Requests can be sent to the Committee’s coordinator, Kelly Zentgraf at zentgraf@upenn.edu, 3535 Market Street, 3rd Floor, Philadelphia, PA 19107, 215–746-6038.

## Abbreviations

BWS: Best-Worst Scaling
CBH: Community Behavioral Health
DBHIDS: Philadelphia Department of Behavioral Health and Intellectual Disability Services
EBP: Evidence-Based Practice
EPIC: Philadelphia Evidence Based Practice and Innovation Center
LCA: Latent Class Analysis
