# Supplementary material for: Community stakeholder preferences for evidence-based practice implementation strategies in behavioral health: a best-worst scaling choice experiment
Source: BMC Psychiatry. 2021 Feb 4;21:74. doi: 10.1186/s12888-021-03072-x (PMC7863375; doi:10.1186/s12888-021-03072-x)
Supplement: Supplementary file 1 — Additional file 1. The BWS prompt and an example set of strategies. [file 12888_2021_3072_MOESM1_ESM.docx]

**Additional file 1**

**Figure 1: Survey Instructions for Best-Worst Scaling Experiment**


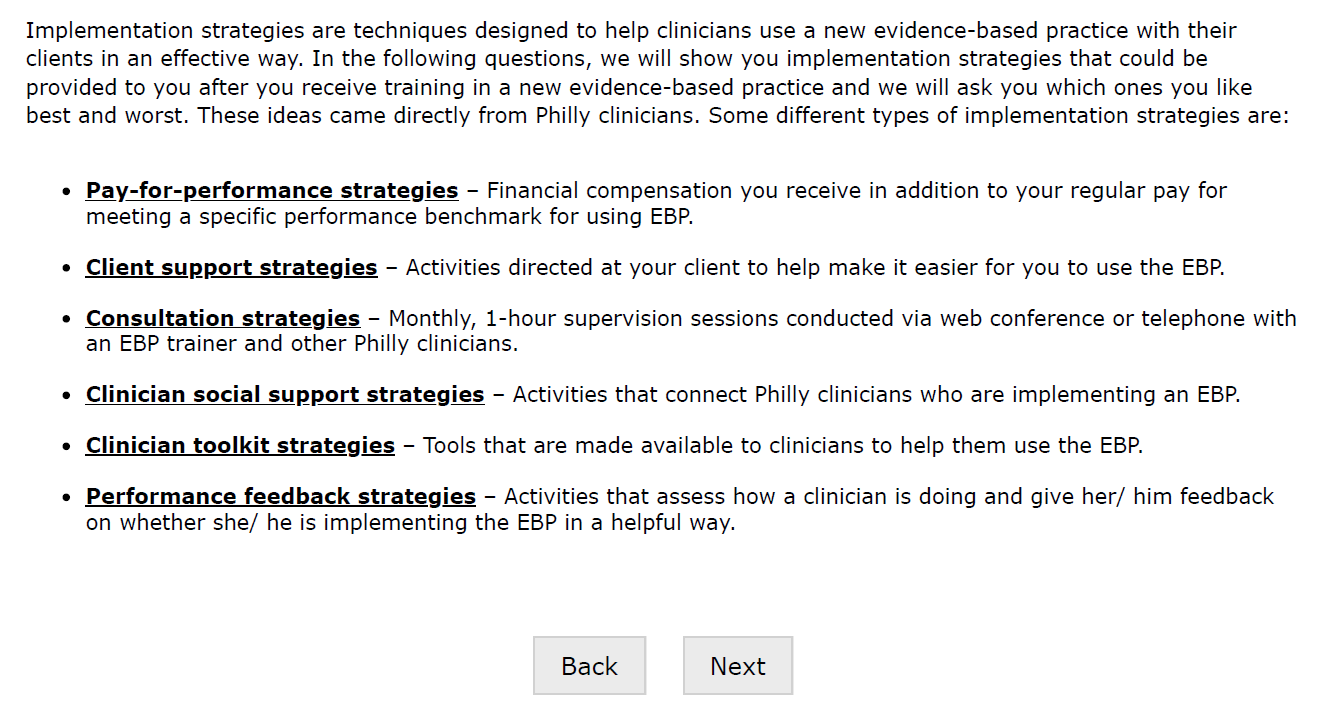


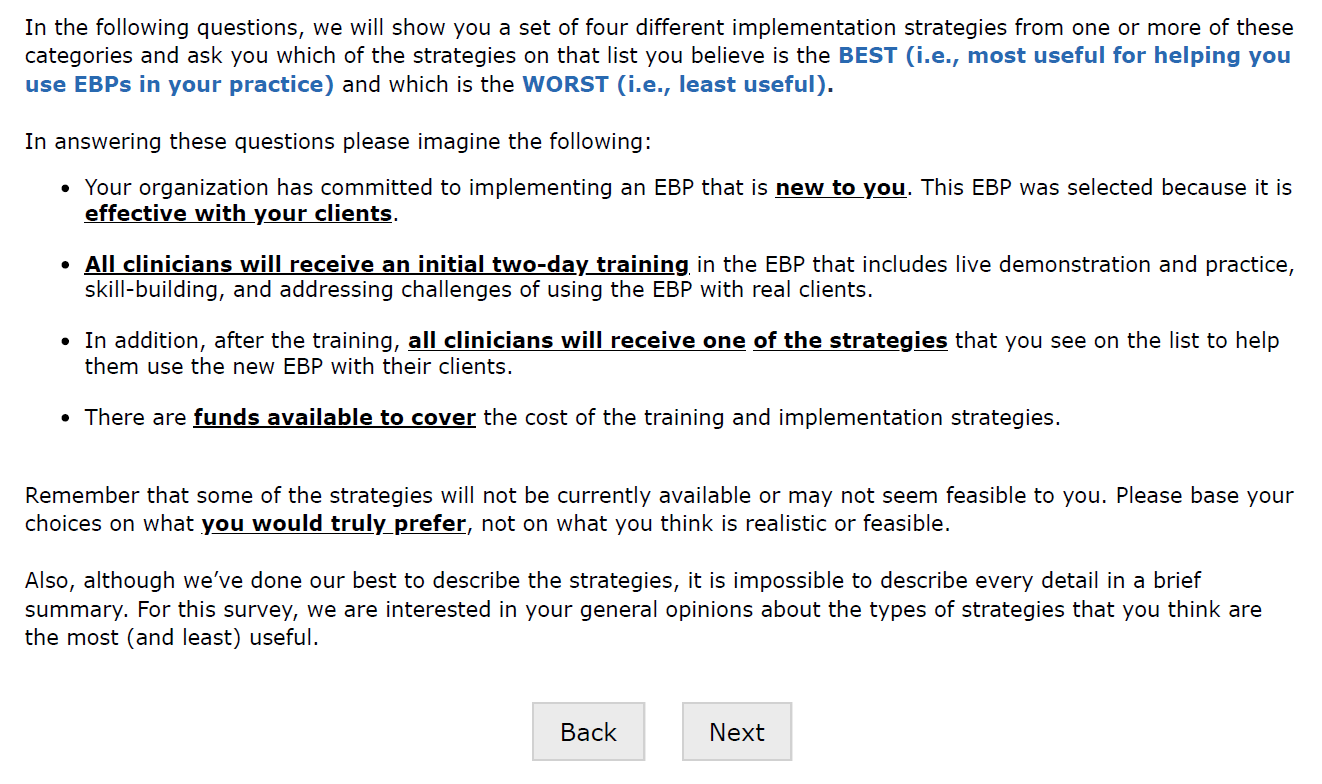


**Figure 2: Example of a Best-Worst Scaling Choice Experiment Question**


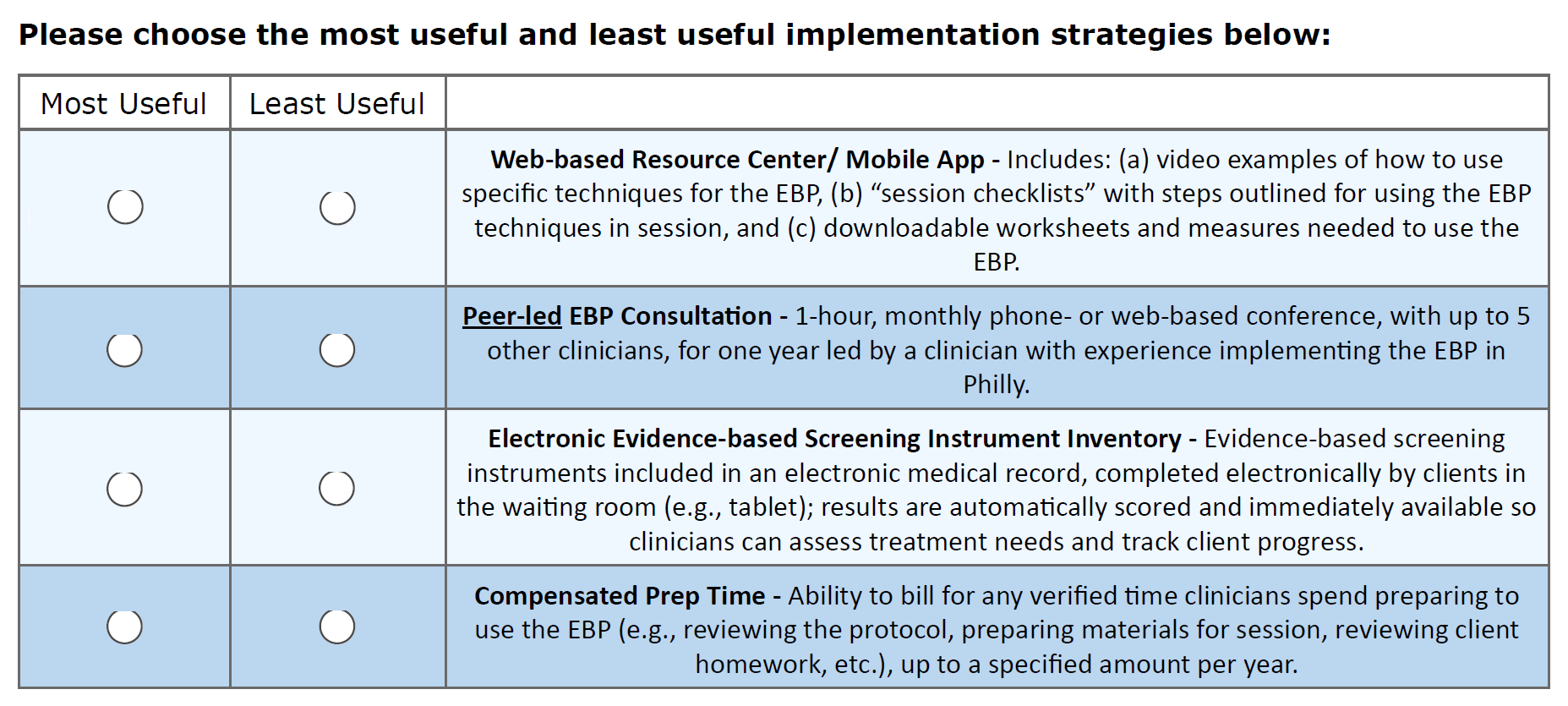


*Note:* This is an example Best-Worst Scaling question. In the experiment, respondents were shown 11 questions which used this format. As is shown here, each question showed 4 of the 14 implementation strategies and asked the respondent to indicate which of those 4 was most useful and which was least useful. The randomization sequence ensured that all respondents viewed all 14 implementation strategies at least 3 times and that the randomization was balanced in terms of (a) position (i.e., which strategy appeared at the top of the list versus at the bottom of the list), and (b) the extent to which each strategy appeared with each of the other strategies.
