# Supplementary material for: Community stakeholder preferences for evidence-based practice implementation strategies in behavioral health: a best-worst scaling choice experiment
Source: BMC Psychiatry. 2021 Feb 4;21:74. doi: 10.1186/s12888-021-03072-x (PMC7863375; doi:10.1186/s12888-021-03072-x)
Supplement: Supplementary file 2 — Additional file 2. Participant Characteristics Overall and by Preference Segment, shows the distribution of professional and sociodemographic characteristics by segment and for the full sample. [file 12888_2021_3072_MOESM2_ESM.docx]

**Table 3.** *Participant Characteristics Overall and by Preference Segment*

|  |  |  | Preference Segment: Support Therapists through… | | | | | | | |  |
| --- | --- | --- | --- | --- | --- | --- | --- | --- | --- | --- | --- |
|  | Total | | ...Financial Incentives | | …Technology | | …Autonomy | | …Consultation | | *p* |
| Characteristic | *n* | % | *n* | % | *n* | % | *n* | % | *n* | % |  |
| Stakeholder Group |  |  |  |  |  |  |  |  |  |  | 0.006 |
| Executive/ Upper management | 29 | 8 | 20 | 17 | 4 | 5 | 2 | 4 | 3 | 3 |  |
| Supervisor of direct providers | 74 | 22 | 28 | 23 | 14 | 18 | 11 | 21 | 21 | 23 |  |
| Direct provider | 240 | 70 | 73 | 60 | 62 | 77 | 39 | 75 | 66 | 73 |  |
| Education |  |  |  |  |  |  |  |  |  |  | 0.262 |
| Doctoral | 39 | 12 | 11 | 9 | 10 | 13 | 8 | 16 | 10 | 12 |  |
| Master's | 278 | 84 | 104 | 89 | 66 | 83 | 37 | 74 | 71 | 83 |  |
| Less than Master's | 14 | 4 | 2 | 2 | 3 | 4 | 5 | 10 | 4 | 5 |  |
| Substance use clinic employee? |  |  |  |  |  |  |  |  |  |  | 0.020 |
| No | 247 | 75 | 90 | 77 | 65 | 82 | 39 | 78 | 53 | 62 |  |
| Yes | 84 | 25 | 27 | 23 | 14 | 18 | 11 | 22 | 32 | 38 |  |
| Salary employee? |  |  |  |  |  |  |  |  |  |  | 0.669 |
| No | 95 | 41 | 28 | 39 | 25 | 41 | 18 | 50 | 24 | 38 |  |
| Yes | 137 | 59 | 44 | 61 | 36 | 59 | 18 | 50 | 39 | 62 |  |
|  |  |  |  |  |  |  |  |  |  |  |  |
|  | *M* | *SD* | *M* | *SD* | *M* | *SD* | *M* | *SD* | *M* | *SD* |  |
| Tenure in agency | 4.30 | 4.80 | 4.80 | 5.49 | 3.09 | 2.81 | 4.98 | 4.55 | 4.32 | 5.23 | 0.061 |
| Years of experience as a clinician | 7.43 | 7.16 | 6.57 | 5.82 | 6.77 | 6.56 | 8.22 | 7.51 | 8.59 | 8.71 | 0.304 |
| Age | 38.65 | 11.19 | 38.70 | 10.81 | 35.97 | 9.76 | 39.68 | 12.53 | 40.49 | 11.83 | 0.065 |
| Research emphasis in graduate school (1 to 7) | 5.18 | 1.49 | 5.10 | 1.55 | 5.07 | 1.52 | 5.38 | 1.53 | 5.25 | 1.39 | 0.711 |
| Ave. hours worked per week | 33.66 | 11.95 | 36.69 | 11.76 | 32.97 | 11.41 | 28.59 | 13.01 | 33.90 | 11.21 | 0.009 |
| N of DBHIDS-sponsored EBP initiatives participated in (1 to 6) | 4.31 | 1.42 | 4.41 | 1.23 | 4.54 | 1.12 | 3.73 | 1.94 | 4.32 | 1.44 | 0.021 |
| N of strategies currently available in agency (1 to 14) | 1.79 | 1.86 | 1.74 | 1.63 | 1.49 | 1.39 | 2.20 | 2.65 | 1.88 | 1.98 | 0.194 |
| Total *N* | 343 | 100 | 121 | 35 | 80 | 23 | 52 | 15 | 90 | 26 |  |

*Note:* Participants were assigned to preference segments via Latent Class Analysis where the best-fitting model was a 4-class solution. DBHIDS = Department of Behavioral Health and Intellectual Disability Services; EBP = evidence-based practice. Mean differences were tested using one-way analyses of variance; differences in proportion were tested using chi-square tests.

**Table 4.** *Distribution of Participants across Preference Segments by Professional Characteristics*

|  |  |  | Preference Segment: Support Therapists through… | | | | | | | |  |
| --- | --- | --- | --- | --- | --- | --- | --- | --- | --- | --- | --- |
|  | Total | | ...Financial Incentives | | …Technology | | …Autonomy | | …Consultation | | *p* |
| Characteristic | *n* | % | *n* | % | *n* | % | *n* | % | *n* | % |  |
| Stakeholder Group |  |  |  |  |  |  |  |  |  |  | 0.006 |
| Executive/ Upper management | 29 | 100 | 20 | 69 | 4 | 14 | 2 | 7 | 3 | 10 |  |
| Supervisor of direct providers | 74 | 100 | 28 | 38 | 14 | 19 | 11 | 15 | 21 | 28 |  |
| Direct provider | 240 | 100 | 73 | 30 | 62 | 26 | 39 | 16 | 66 | 28 |  |
| Education |  |  |  |  |  |  |  |  |  |  | 0.262 |
| Doctoral | 39 | 100 | 11 | 28 | 10 | 26 | 8 | 20 | 10 | 26 |  |
| Master's | 278 | 100 | 104 | 37 | 66 | 24 | 37 | 13 | 71 | 26 |  |
| Less than Master's | 14 | 100 | 2 | 14 | 3 | 21 | 5 | 36 | 4 | 29 |  |
| Substance use clinic employee? |  |  |  |  |  |  |  |  |  |  | 0.020 |
| No | 247 | 100 | 90 | 36 | 65 | 26 | 39 | 16 | 53 | 22 |  |
| Yes | 84 | 100 | 27 | 32 | 14 | 17 | 11 | 13 | 32 | 38 |  |
| Salary employee? |  |  |  |  |  |  |  |  |  |  | 0.669 |
| No | 95 | 100 | 28 | 30 | 25 | 26 | 18 | 19 | 24 | 25 |  |
| Yes | 137 | 100 | 44 | 32 | 36 | 26 | 18 | 13 | 39 | 27 |  |
| Total *N* | 343 | 100 | 121 | 35 | 80 | 23 | 52 | 15 | 90 | 26 |  |

*Note:* These data are identical to Table 3 but rather than reporting the percentage of each segment that belongs to each level of the categorical variable, this table presents the percentage of each level of each categorical variables (e.g., Executives/Upper Management) that belongs to each segment. DBHIDS = Department of Behavioral Health and Intellectual Disability Services; EBP = evidence-based practice.

**Supplemental Table 1A.** Crosswalk of implementation strategies from present study with those generated by the ERIC project.

| Category | Strategy Name | Definition | Related strategy from ERIC |
| --- | --- | --- | --- |
| Financial Incentives | EBP certification bonus | Receipt of a 1-time bonus for verified completion of a certification process over a 1-year period, in which clinicians: attend four, 1-day booster training sessions; pass a multiple-choice knowledge test; and submit one tape of a session with a client where they use the EBP. | - Alter incentive structures |
|  | Compensation for use of EBP per session | Receipt of additional compensation (in addition to regular paycheck) upon verification of using the EBP in sessions with clients for whom it is appropriate (i.e., per session), up to a specified amount per year. | - Alter incentive structures |
|  | Compensated time for preparation | Ability to bill for any verified time clinicians spend preparing to use the EBP (e.g., reviewing the protocol, preparing materials for session, reviewing client homework, etc.), up to a specified amount per year. | - Alter incentive structures |
| Clinical Consultation | Expert-led EBP consultation | 1-hour, monthly, web- or phone-based consultation, with up to 5 other clinicians, for one year led by an expert EBP trainer. | - Clinical supervision - Use an implementation advisor - Provide ongoing consultation |
|  | Peer-led EBP consultation | 1-hour, monthly web- or phone-based conference, with up to 5 other clinicians, for one year led by a clinician with experience implementing the EBP in Philadelphia. | - Clinical supervision - Local technical assistance - Use train-the-trainer strategies |
|  | Expert in your back pocket (on call) | Network of EBP trainers on call via phone or web chat for same-day, 15-minute consultations to problem-solve issues with implementing the EBP. | - Clinical supervision - Use an implementation advisor - Provide ongoing consultation |
| Clinical Support Tools | Web-based resource center/ mobile app | Includes: (a) video examples of how to use specific techniques for the EBP, (b) “session checklists” with steps outlined for using the EBP techniques in session, and (c) downloadable worksheets and measures needed to use the EBP. | - Distribute educational materials |
|  | Electronic evidence-based screening instrument inventory | Evidence-based screening instruments included in an electronic medical record, completed electronically by clients in the waiting room (e.g., tablet); results are automatically scored and immediately available so clinicians can assess treatment needs and track client progress. | - Facilitate relay of clinical data to providers - Change record systems |
| Clinician Social Support and Networking | EBP-focused online forum | Confidential site available only to registered clinicians who use the EBP, where clinicians can login and post questions and answers about using the EBP, share tips, and identify resources for using the EBP. | - Promote network weaving - Create a learning collaborative |
|  | Community-based EBP mentoring program | One-on-one mentoring program, where clinicians are matched with a local peer clinician who works with the same population to support each other in implementing the EBP. | - Promote network weaving - Create a learning collaborative |
| Performance Feedback / Social Comparison | EBP Performance benchmark leaderboard | Posted where only agency staff can view it, recognizing clinicians in the agency who met a benchmark for EBP implementation each month (based on 3 randomly selected sessions). | - Audit and feedback |
|  | EBP Performance benchmark email | Available only to the clinician and his/her supervisor, reporting whether s/he met a benchmark for EBP implementation each month (based on 3  randomly selected sessions). | - Audit and feedback |
| Client Supports | Client mobile app/ texting service | Provides clients with reminders to attend sessions, prompts to complete homework assignments, and clinician-tailored messages about practicing EBP skills. | - Intervene with patients to enhance uptake and adherence |
|  | Improved waiting room | Create a relaxing waiting room (e.g., physical appearance, sensory experience) that helps prepare the client to enter the session ready to work on EBP content. | - Prepare patients to be active participants - Change physical infrastructure and equipment |

*Note:* ERIC = Expert Recommendations for Implementing Change, see Powell et al., 2015, A refined compilation of implementation strategies: results from the Expert Recommendations for Implementing Change (ERIC) project, *Implementation Science, 10*(21), doi: 10.1186/s13012-015-0209-1.
